# Evaluating short-chain fatty acids in breath condensate as surrogate measurements for systemic levels and investigation into alternative respiratory sample matrices

**DOI:** 10.1042/CS20257925

**Published:** 2025-11-04

**Authors:** Christopher G. Green, Joycelyn Bempong, Marilyn L.Y. Ong, Anand Shah, Patrick Mallia, Sebastian L. Johnston, Aran Singanayagam, James C. Reynolds, Liam M. Heaney

**Affiliations:** 1 School of Sport, Exercise and Health Sciences, Loughborough University, Loughborough, U.K; 2 Department of Chemistry, School of Science, Loughborough University, Loughborough, U.K; 3 Exercise and Sports Science Programme, School of Health Sciences, Health Campus, Universiti Sains Malaysia, Kelantan, Kubang Kerian, 16150, Malaysia; 4 Centre for Bacterial Resistance Biology, Imperial College London, London, U.K; 5 National Heart and Lung Institute, Imperial College London, London, U.K

**Keywords:** Biomarker, breath, gut, postbiotics, SCFAs, lung microbiome

## Abstract

Short-chain fatty acids (SCFAs) are metabolic by-products from microbial fermentation of complex carbohydrates and protein. They have gained clinical interest for their protective effects, including within the lung microenvironment. SCFAs are detectable in circulation and exhaled breath condensate (EBC), posing questions as to whether exhaled SCFAs originate from the gut and/or lung microbiota. Mapping SCFAs from the lung could improve our understanding of microbial activity in respiratory conditions. SCFA measurements in EBC were evaluated using a validated gas chromatography-mass spectrometry assay. Six healthy participants ingested sodium acetate, calcium propionate and sodium butyrate to acutely increase circulating SCFAs. EBC samples were collected alongside venous draws, with circulating and exhaled levels compared. A series of additional respiratory sample matrices from patient samples was investigated to gain novel insights into SCFAs within different respiratory biofluids. Serum SCFAs were increased in line with known responses. However, these increases were not observed in EBC, indicating a lack of correlation between circulating and exhaled SCFAs. SCFAs were detected in all additional respiratory biosamples, with EBC and sputum reporting the highest concentrations. Interestingly, branched-chain moieties were notably abundant in sputum, indicating the potential for their local production by bacterial fermentation of lung mucus proteins. SCFAs in EBC do not reflect circulatory levels and, therefore, are not a suitable surrogate measurement to inform on systemic load. These data suggest that exhaled SCFAs are potentially derived from lung microbial metabolism, supporting the need for further investigation into SCFA production, function and diagnostic utility in respiratory health.

## Introduction

The measurement of endogenous biological markers, or biomarkers, is routine practice within both clinical and research environments. The measurement of disease-associated markers can help to improve diagnostic, prognostic and therapeutic monitoring of conditions [1]. In general, venous blood samples are the biofluid of choice (as whole blood, plasma or serum) to measure and quantitate biomarkers. However, venous blood draw collections require an invasive approach using a sharpened needle to puncture the skin and vein wall. In addition, trained personnel are required to perform the procedure, and thus the collections are usually hospital/clinic based or require the phlebotomist to travel to the location of the patient [2]. It is, therefore, of interest for clinical protocols to assess the potential to collect biofluid samples that require less invasive and technically demanding methods to improve the biomarker sampling workflow for both clinicians and patients alike [2]. One biofluid collection which has garnered attention is exhaled breath condensate (EBC). EBC is collected as the result of condensing exhaled vapours and aerosols into an aqueous liquid which contains volatile and non-volatile chemical species ejected from the lung during exhalation. This biofluid offers clinicians and researchers the opportunity to monitor specific biomarker characteristics using a minimally invasive technique [3]. For example, EBC samples offer the ability to provide insight into the happenings/condition of the lung microenvironment, as well as providing information on molecules present in systemic circulation that can cross the blood–alveolar barrier; consequently, they offer the potential to identify valuable information regarding both respiratory and systemic health with minimal discomfort to the patient [4].

It has been recently demonstrated that short-chain fatty acids (SCFAs) are detectable within EBC samples [5]. These molecules are short (C2-C5), saturated carboxylic acids that have been shown to be associated with protective roles in the context of health and disease [6,7]. SCFAs are produced as by-products from the fermentation of dietary fibre and other non-digestible carbohydrates by the gut microbiota [8]. These degradation processes form straight-chain acids of which the most abundant are acetic acid (C2), propionic acid (C3) and butyric acid (C4), with smaller amounts of valeric acid (C5) produced. To note, acetic acid (in its dissociated form as acetate) is also produced endogenously via the hydrolysis of acetyl-CoA in the liver [9]. In addition, bacterial breakdown of proteins can lead to the production of the branched-chain species isobutyric acid (C4), isovaleric acid (C5) and 2-methylbutyric acid (C5) [10]. However, as these molecules are produced within the intestinal environment, only a small proportion of these bacterially derived SCFAs reach peripheral circulation [11]. Importantly, in the context of health and disease, SCFAs have been shown to possess anti-inflammatory properties as well as being capable of impacting metabolic processes related to substrate utilisation and appetite hormone secretion [12–15], although their impact within the intestine has also been shown to exert pathological effects [16]. The effects of SCFAs within the respiratory system are poorly defined; however, there is accumulating evidence that SCFAs play an important role in respiratory health via the gut-lung axis [17,18]. Specifically, SCFAs may have a role in reducing susceptibility and/or improving outcomes to various respiratory diseases (e.g., SARS-CoV-2, influenza virus and respiratory syncytial virus infection) [19–25] and regulating the inflammatory response to non-communicable conditions (e.g., asthma, allergy) [26,27]. Conversely, SCFAs have been shown to induce pro-inflammatory responses in diseased lung cells (cystic fibrosis) as well as supporting the growth of the common lung colonising bacteria *Pseudomonas aeruginosa* [28]. Interestingly, *in vitro* work assessing the impact of butyrate on human macrophages reported a biphasic approach where lower concentrations exerted an anti-inflammatory response and higher concentrations a pro-inflammatory response [29]. Therefore, SCFAs may have divergent roles in different contexts within the lungs, providing an avenue of interest to better understand concentrations of SCFAs as potential biomarkers of health and disease. In this light, measurement of blood fluids (i.e. plasma/serum) is the currently accepted approach for the quantitation of SCFAs that are available at the systemic level [11]. Whilst quantitation of SCFAs in faeces is commonly performed, the values measured are not suggested to be representative of systemically available SCFAs and, therefore, not (directly) physiologically active outside the intestinal microenvironment [11]. However, the availability of SCFAs in the lungs is of particular interest given the links identified for protection/improvement in respiratory health. SCFAs present in the lung can be partly explained by the gut-lung axis through the translocation from the colon into systemic circulation followed by transfer into the lung microenvironment via the blood–alveolar barrier [17,30]. In addition, our knowledge surrounding the respiratory microbiome is ever-growing and therefore it is of great interest to better understand the potential for SCFAs to be produced locally within the lung [31].

Importantly, the measurement of SCFAs in respiratory biosamples offers the potential for a minimally invasive approach to act as a surrogate measure for circulating SCFAs, as well as an opportunity to compare blood and breath values to enrich our understanding of the gut–lung axis and to investigate contributory aspects of the respiratory microbiota. Therefore, this project aimed to assess the use of EBC sampling to understand the relationship between systemic and exhaled SCFAs. To achieve this comparison, an induced rise in circulating SCFAs was performed via oral SCFA consumption with serum and EBC samples collected across a 2 h time course. In addition, a series of clinical respiratory samples were investigated to understand the presence and distribution of SCFAs in lung-derived biofluid samples.

## Methods

### Validation of quantitative measurement of SCFAs in EBC

#### Materials

Hydrochloric acid (1M) and LC-MS grade water were purchased from VWR Chemicals (Lutterworth, U.K). Methyl-tert-butyl ether (MTBE) (99.9% purity) was purchased from Acros Organics (Loughborough, U.K). Acetic acid (≥99.99% purity), propionic acid (≥99.5% purity), isobutyric acid (≥99.5% purity), butyric acid (>99% purity), isovaleric acid (>99% purity), 2-methylbutyric acid (>99% purity) and valeric acid (≥99.8% purity) were purchased from Merck (Gillingham, U.K). Acetic acid-d4 (≥99.5% purity, >95% isotopic enrichment) was purchased from Alfa Aesar (Heysham, U.K), isobutyric acid-d3 (98.7% purity, >95% isotopic enrichment) was purchased from QMX (Thaxted, U.K), propionic acid-d3 (98% purity, 99.6% isotopic enrichment), butyric acid-d7 (98% purity, 99.3% isotopic enrichment), isovaleric acid-d9 (98% purity, 97.9% isotopic enrichment), 2-methylbutyric acid-d3 (99% purity, 99.3% isotopic enrichment) and valeric acid-d9 (98% purity, 98.6% isotopic enrichment) were purchased from Toronto Research Chemicals (Toronto, Canada).

#### SCFA analyses

SCFA concentrations were measured by quantitative gas chromatography-mass spectrometry (GC-MS) using a validated assay [32]. An Agilent 7820A GC system (Agilent Technologies, Stockport, U.K) fitted with a Stabilwax-DA Crossbond Carbowax PEG column (30 m × 0.5 mm × 0.25 μm; Thames Restek, High Wycombe, U.K) was used. The GC system was coupled with a 5977B MSD single quadrupole mass analyser (Agilent Technologies). In brief, 100 µl of sample was mixed with 100 µl 1M hydrochloric acid and 100 µl of an internal standard (IS) mixture. The IS contained 6 µg/ml of each deuterium-labelled SCFA in MTBE. Each sample was vortexed for 30 s prior to centrifugation at 5400 ***g*** for 15 min at 15°C. The organic layer (~50 µl) was transferred to a low-volume autosampler vial for analysis. All samples, including three quality control (QC) samples at low (100 ng/ml), medium (200 ng/ml) and high (375 ng/ml) concentrations, were placed in a randomised sequence for analysis. QC samples for acetic acid were at a concentration of 10× that of the other SCFAs due to higher basal levels.

Each sample was analysed in duplicate (3 µl of sample per injection) with ~12.5 min between injections. The inlet and transfer line temperatures were set to 250°C. The electron ionisation source temperature was set to 230°C and had a fixed ionisation energy of 70 eV applied. The quadrupole mass analyser temperature was set to 150°C. Purified helium at a constant flow rate of 2 ml/min was used as a carrier gas. The GC oven was programmed with a double ramp temperature increase protocol. The initial temperature was set to 80°C for 1 min before linearly increasing to 127°C at a rate of 10°C/min. From this point, the oven temperature increased linearly at a rate of 30°C/min until a temperature of 181°C. The run time was 7.5 min, followed by a post-run temperature hold of 2 min at 230°C. To ensure no carryover of samples, a blank run (100% MTBE) was implemented after every duplicate injection. The MSD was set to scheduled selected ion monitoring mode. MassHunter software (Version B.07.00; Agilent Technologies, Stockport, U.K) was used for GC-MS data acquisition and monitoring.

#### EBC collection

**Figure 1:**
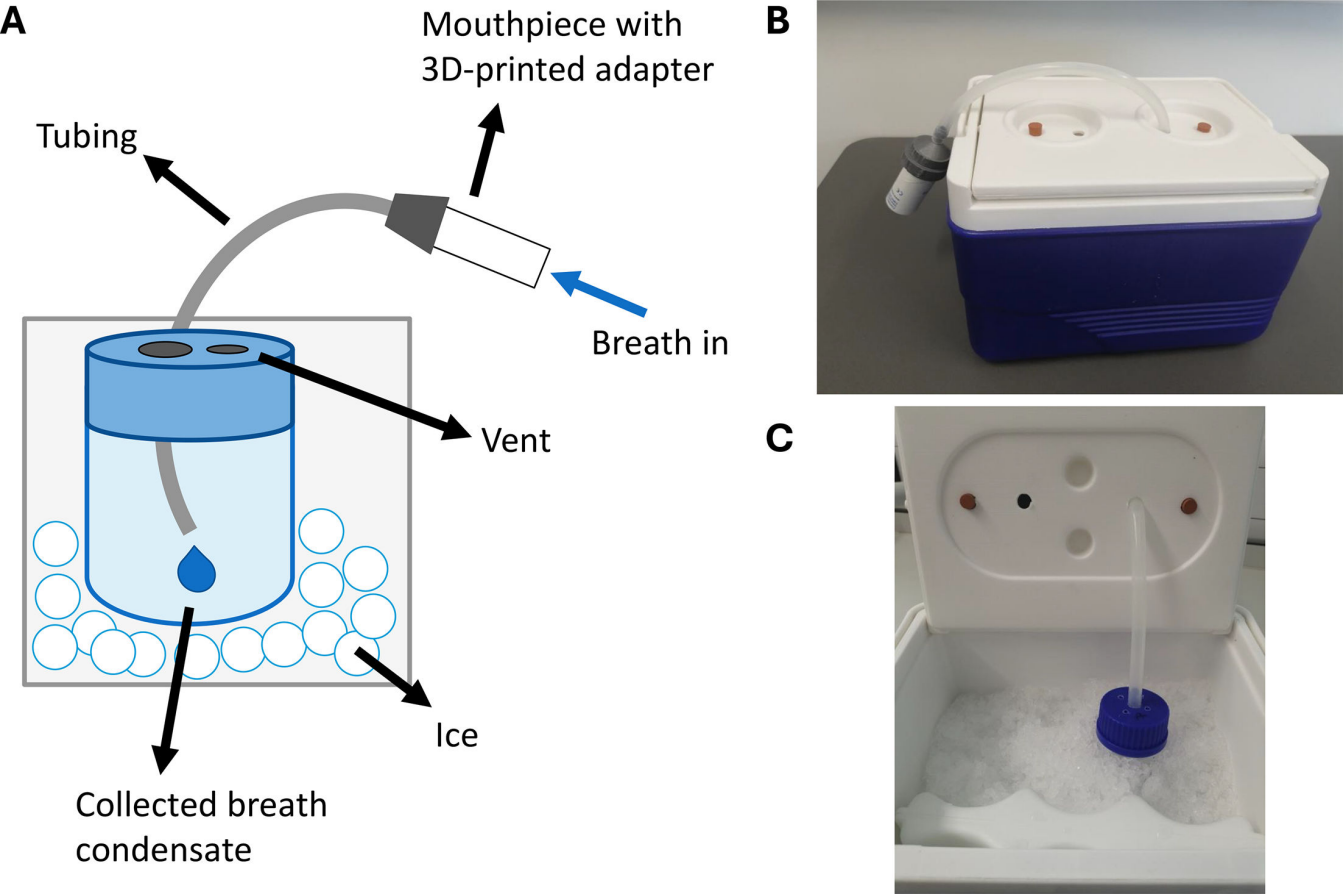
Details of the custom-built exhaled breath condensate sample collector, including (**A**) a schematic diagram of sample collector components, (**B**) an outside view of the sample collector with mouthpiece and sampling tubing connected, and (**C**) an inside view of the sample collector with the collection bottle submerged in ice.

EBC samples were collected using a custom-built sampling device (Figure 1). This consisted of a disposable cardboard mouthpiece (Clement-Clarke International, Mountain Ash, U.K) attached to a 40 cm long silicone tube (10 mm outer diameter, 6 mm internal diameter) via a 3D-printed polylactic acid filament adapter. The opposite end of the silicone tubing was attached to a 5 ml collection bottle (DURAN®, DWK Life Sciences, Mainz, Germany) via port holes in the lid of a plastic insulated cool box containing ice. For all EBC collections, participants were asked to cover the mouthpiece entirely and continue breathing in a normal pattern for 5 min. If a low volume of EBC was present within the collection bottle at the cessation of the 5 min collection period (<1 ml), participants were asked to continue breathing through the mouthpiece for a further 5 min, or until a sufficient volume of EBC (approximately 2 ml) was produced. Once sampling was complete, 1 ml aliquots of the EBC were transferred to microcentrifuge tubes and stored at –80°C until analysis. Each collection of SCFAs in this experiment used new, clean silicone tubing which was sanitised in sterilising fluid following use (Milton Disinfecting Fluid, Proctor & Gamble, Cincinnati, OH, U.S.A.).

#### Matrix effect and analyte recovery for measurement of SCFAs in EBC

The matrix effect and recovery of SCFAs in EBC were performed as described previously [32]. An SCFA stock solution was prepared that contained the SCFAs dissolved in water at a concentration of 4000 ng/ml, with the exception of acetic acid, which was present at 40,000 ng/ml due to higher basal levels. A pooled EBC sample was prepared by combining and mixing a random selection of EBC samples used as part of the experiment described later. Pooled EBC samples were mixed with the SCFA stock solution at different ratios to create 100 µl standards at 0 (i.e. pooled EBC mixture), 400/4000 (for C3+ SCFAs/acetic acid), 800/8000, and 1600/16,000 ng/ml, representing a blank, low, medium and high standard, respectively. Four SCFA standards matched for concentration were also produced in LC-MS water.

To determine the matrix effect, the difference between the gradients for SCFA concentration in EBC and water standards was compared [33]. To assess analyte recovery, the observed concentration measured for each spiked standard was compared with the expected concentration calculated based on the ratio of pooled EBC and spiked standard present in each analysed mixture.

### Comparison of SCFAs in serum and EBC

#### Participants

Six healthy male participants [mean (range), 31 (25–38) yrs, 87 (73–101) kg] volunteered to take part in the experiment, provided written informed consent to take part, and were free to withdraw at any point. All procedures were approved by the Loughborough University Ethics Review Sub-Committee (Human Participants, approval ref: 6688). All participants completed one experimental trial.

#### SCFA supplements

Anhydrous sodium acetate (NaAc) and calcium propionate (CaPr) were purchased from Merck (both Food Chemical Codex grade, Merck). Sodium butyrate (NaBu) supplements were purchased from BodyBio (BodyBio, Millville, NJ, U.S.A.). NaAc and CaPr were encapsulated in opaque size 000 VCaps® Plus (Lonza, Basel, Switzerland) made from hydroxypropyl methylcellulose designed for quick release within the gastrointestinal tract [34].

#### Supplementation experiment protocol

Participants were asked to attend the laboratory during the morning and to not have consumed any food or drink other than plain water or to have brushed their teeth in the hour prior to arrival. Upon arrival, participants completed 10 min of seated rest before a cannula was placed into a vein in the antecubital fossa and a baseline blood sample was drawn. A baseline EBC collection was started as soon as possible following the blood collection. Participants then ingested 3 g of NaBu and 2 g each of NaAc and CaPr with plain water as quickly as was comfortable (<10 min). Further blood and EBC samples were collected at 30-, 60- and 120 min post-ingestion. The 120 min investigation period was selected based on previous work demonstrating the described supplementation protocol showing peak circulating values within 60 min and a return to baseline values by 120 min [34].

#### Blood handling and sample storage

All blood collections were drawn into serum clotting activator tubes (S-Monovette®, Sarstedt, Nümbrecht, Germany) and placed on ice for 30 to 45 min before centrifugation at 2500 ***g*** for 20 min at 4°C. Serum and EBC samples were aliquoted into microcentrifuge tubes and stored at –80°C until analysis.

#### Patient respiratory biosamples

In addition to EBC samples collected for the supplementation experiment, a series of banked clinical respiratory biosamples from historical studies were investigated for SCFA content. These samples were collected as part of prior studies evaluating subjects with chronic obstructive pulmonary disease (COPD), smokers without COPD, healthy non-smoking controls and patients sampled following discharge after hospitalised COVID-19. Full details of patient demographics and information regarding ethical approval can be found within the original manuscripts describing these studies [35–37]. The biofluids analysed included EBC (*n*=36), sputum (*n*=36), nasal lavage fluid (*n*=56), bronchoalveolar lavage fluid (BAL, *n*=57), and nasal lining fluid (*n*=81). Specifically, EBC was collected using a commercial ECoScreen EBC-Collector (FILT Lungen- und Thoraxdiagnostik GmbH, Berlin-Buch, Germany). Sputum was induced by inhalations of hypertonic (4%) saline administered with an UltraNeb99 ultrasonic nebuliser (Drive DeVilbiss Healthcare, Halifax, U.K) and processed using standard protocols [35,36]. Nasal lavage fluid sampling was performed by instilling 2.5 ml of 0.9% saline into each nostril, holding for 5 s and then expelling into a sterile container, followed by sample homogenisation [35]. BAL was obtained by bronchoscopy and collected by instillation of sterile 0.9% saline into the left upper lobe bronchus in 30 ml aliquots to a total of 240 ml [35]. Nasal lining fluid was collected through the application of synthetic absorptive matrices to absorb fluid lining of the inferior turbinate [38]. For the purposes of this investigation, samples were collated by biofluid type and not investigated for SCFA levels across sample origin.

#### Statistical analyses

Statistical analyses were performed using STATA MP (v17, StataCorp, Texas, U.S.A.), IBM SPSS (v28, IBM, Illinois, U.S.A.), and R Studio (v2025.05.1, Posit PBC, Vienna, Austria; R Statistics v4.5.0). One-way linear mixed-effects models were used to analyse SCFA concentrations in serum and EBC across the 120 min collection period with random intercepts by participant. Individual models were computed for each biofluid type using the restricted maximum-likelihood estimation with small sample inference on time-by-trial interaction using the Kenward–Roger degrees of freedom method. Where a difference and/or interaction was reported, post-hoc contrasts were performed and adjusted for multiple comparisons using a false discovery rate (FDR) of 5% following the Benjamini–Hochberg method [39]. All post-hoc comparison data are reported as the raw *P* value alongside the critical value (V_CRIT_) calculated as part of the Benjamini–Hochberg FDR procedure. Comparisons where the *P*-value is greater than the V_CRIT_ demonstrate that any difference observed did not satisfy the correction for multiple comparisons. Paired sample t-tests were performed to assess comparisons between baseline (i.e. pre-ingestion) values of acetic acid and butyric acid in serum and EBC, with comparisons for propionic acid performed using the Wilcoxon rank-sum test due to a skewed data distribution. A repeated measures correlation coefficient test was performed using the *rmcorr* package for R Statistics to assess the relationship between SCFA concentrations in serum and EBC at all timepoints. All data are presented as mean (standard error of the mean, SEM) unless otherwise stated. An alpha value was set at *P*<0.05.

## Results

### Matrix effect and analyte recovery for measurement of SCFAs in EBC

To assess the validity of measuring SCFAs in EBC using a quantitative GC-MS method previously validated for serum, plasma and urine [32], matrix effects and analyte recovery data were assessed for the full panel of measured SCFAs. No significant matrix effects were observed in 6/7 SCFA analytes (range 90–110%, Table 1), with only propionic acid showing analyte suppression, reflected by a matrix effect of 65%. The levels of recovery were considered acceptable across all SCFAs, with only 6/21 assessments giving a value outside of 100% ± 10%. Slightly reduced analyte recovery was seen for isobutyric acid in the low (76%) and medium (86%) standards, with butyric acid (88%), isovaleric acid (88%) and valeric acid (86%) showing slightly reduced recovery in the low standard. Acetic acid was the only analyte to report a slightly increased analyte recovery in the high standard (117%).

**Table 1:** Matrix effect and recovery values for seven quantitated short-chain fatty acids in exhaled breath condensate.

		Recovery		
	Matrix effect (**%)**	High standard (**%)**	Medium standard (**%)**	Low standard (**%)**
Acetic acid	90	117	103	102
Propionic acid	65	107	99	98
Isobutyric acid	110	98	86	76
Butyric acid	105	102	93	88
2-Methylbutyric acid	108	103	94	91
Isovaleric acid	97	101	92	88
Valeric acid	106	100	91	86

### Supplementation experiment

To understand the comparative values of SCFAs in the EBC and serum, investigations into paired differences in basal SCFA levels were performed. The baseline concentration of acetic acid in EBC [mean (range), 14,043 (8022–17,825) ng/ml] was greater than serum [3652 (2205–6972) ng/ml] (*P*=0.006). The baseline concentration of propionic acid in EBC [1012 (506–1336) ng/ml] was greater than serum [74 (21–105) ng/ml] (*P*=0.028). The baseline concentration of butyric acid in EBC [33 (0–81) ng/ml] was not different from serum [24 (0–67) ng/ml] (*P*>0.05).

**Figure 2:**
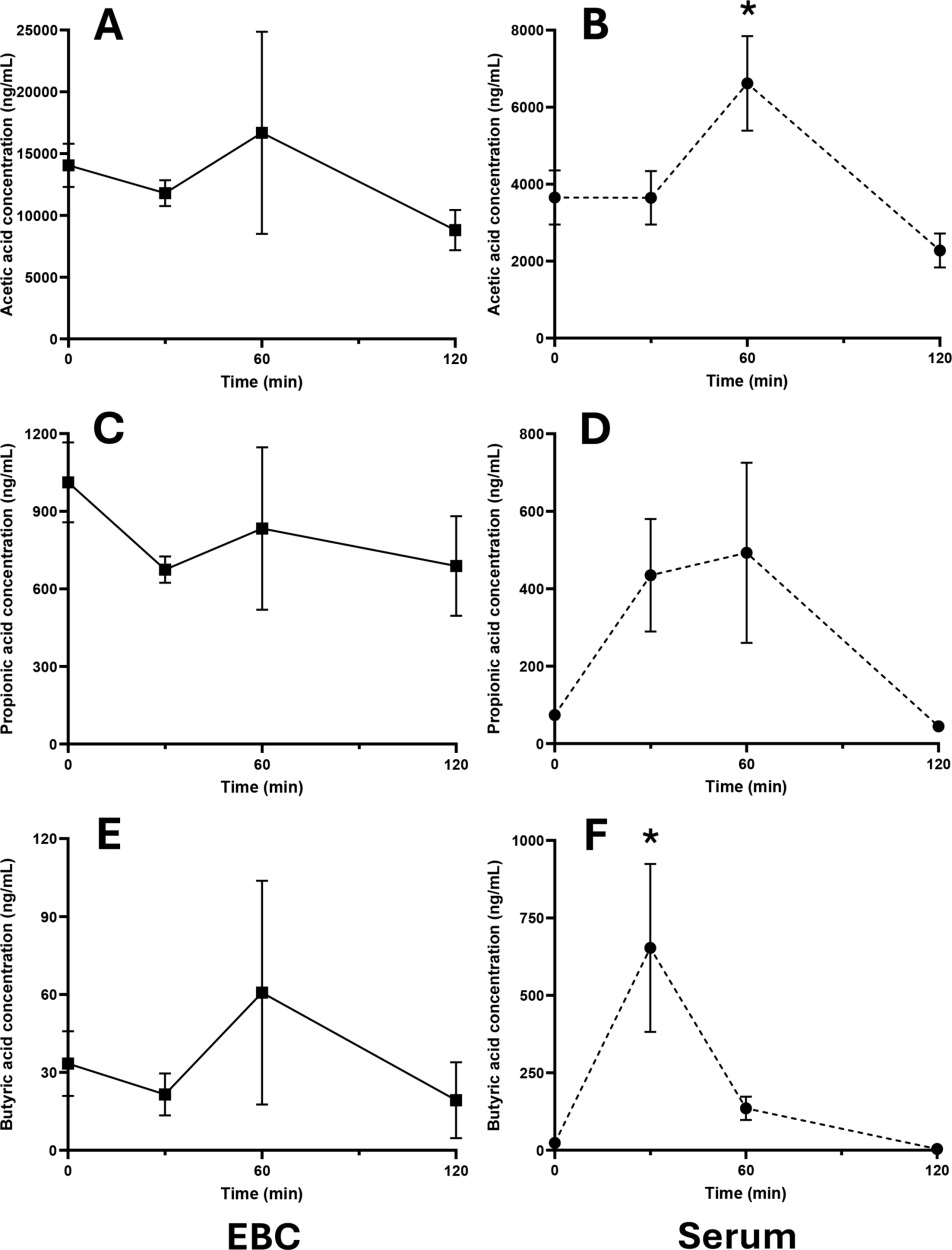
Concentrations of acetic acid, propionic acid and butyric acid within exhaled breath condensate (EBC) (**A, C, E**) and serum (**B, D, F**) in ng/ml following supplementation. Squares indicate mean EBC concentration and circles indicate mean serum concentration. Error bars visualise the standard error of the mean (*n*=6). *=different from baseline (all, *P*<0.05).

To assess the influence of oral supplementation on SCFAs in EBC and serum, biofluid concentrations were compared within both sample mediums at baseline 30-, 60- and 120-min post supplementation (Figure 2). The concentration of acetic acid in EBC was not different from baseline at any timepoint following supplementation. Serum acetic acid concentration was not different compared with baseline at 30 min but was greater at 60 min (*P*=0.010, *V_CRIT_* =0.017), returning to baseline levels by 120 min. The concentration of propionic acid in EBC was not different from baseline at any timepoint following supplementation despite a visual increase in mean concentrations at 30 and 60 min, with comparisons for 60 min and baseline violating the 5% FDR post-hoc correction (*P*=0.026, *V_CRIT_* =0.017). The concentration of butyric acid in EBC was not different from baseline at any timepoint following supplementation. Serum butyric acid concentration was greater at 30 min compared with baseline (*P*=0.001, *V_CRIT_* =0.008) and returned to baseline levels by 60 min.

**Figure 3:**
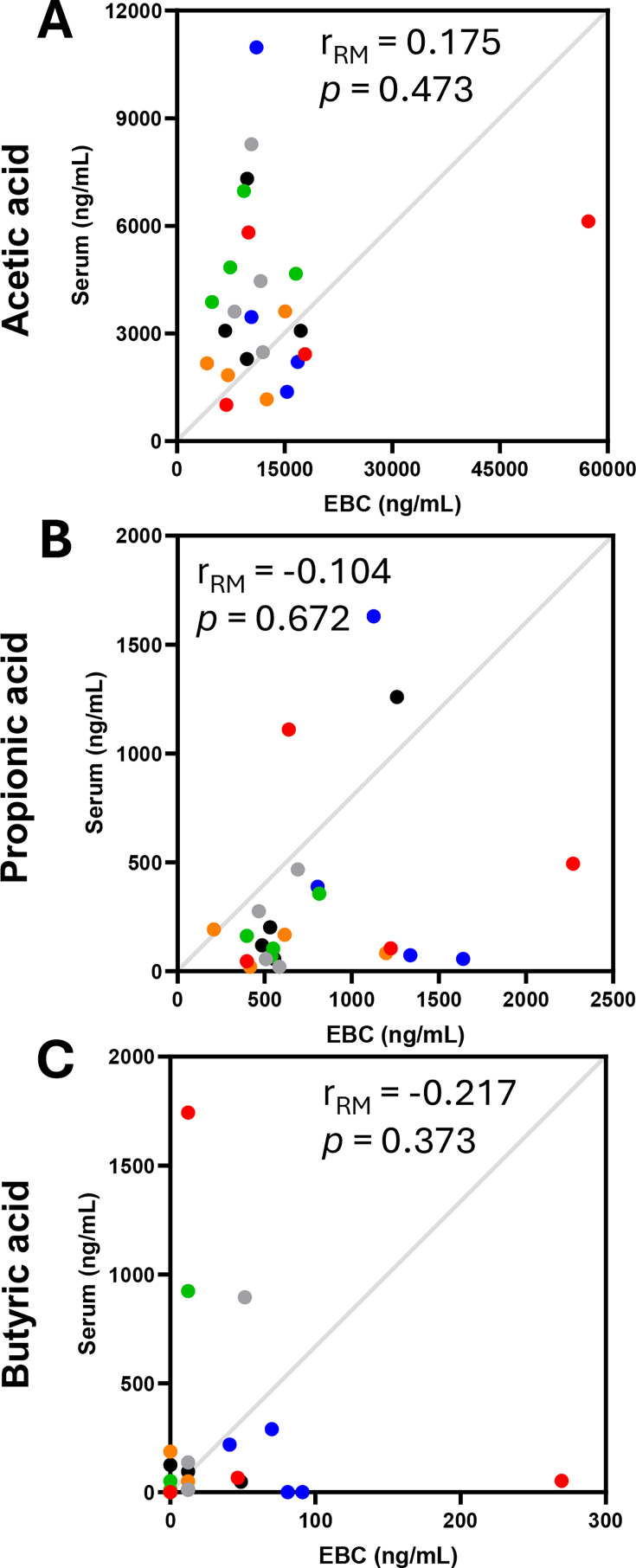
Repeated measures correlations between exhaled breath condensate (EBC) and serum concentrations of acetic acid (**A**), propionic acid (**B**) and butyric acid (**C**) collected at all four measurement timepoints following supplementation (*n*=6). Each participant is represented by a single colour. r_RM_=repeated measures correlation coefficient.

To understand whether SCFA levels in EBC were correlated to those in serum, repeated measures correlation tests were performed. No correlations were seen between EBC and serum SCFA concentrations when the data were assessed across all timepoints (Figure 3).

#### Patient respiratory biosamples

**Figure 4:**
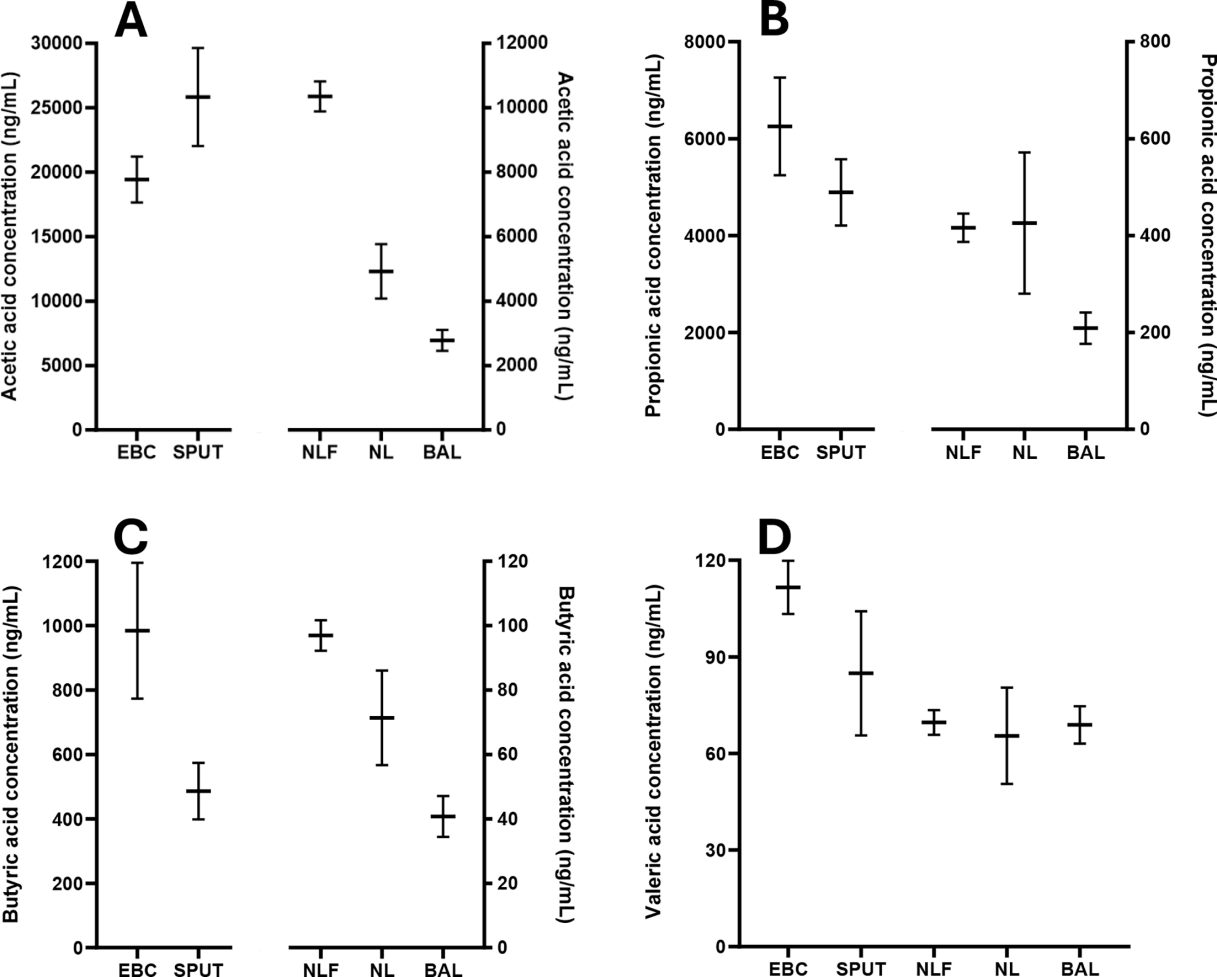
Concentrations of the straight-chain, short-chain fatty acids acetic acid (**A**), propionic acid (**B**), butyric acid (**C**) and valeric acid (**D**) within clinical respiratory samples. Horizontal bars represent the mean concentration with error bars visualising the standard error of the mean. BAL, bronchoalveolar lavage (*n*=54, 56, 46 and 49); EBC, exhaled breath condensate (*n* of samples detected in=acetic, propionic, butyric and valeric; 35, 33, 36 and 35); SPUT, sputum (*n*=36, 36, 32 and 10); NL, nasal lavage fluid (*n*=56, 56, 52 and 18); NLF, nasal lining fluid (*n*=77, 80, 81 and 81).

**Figure 5:**
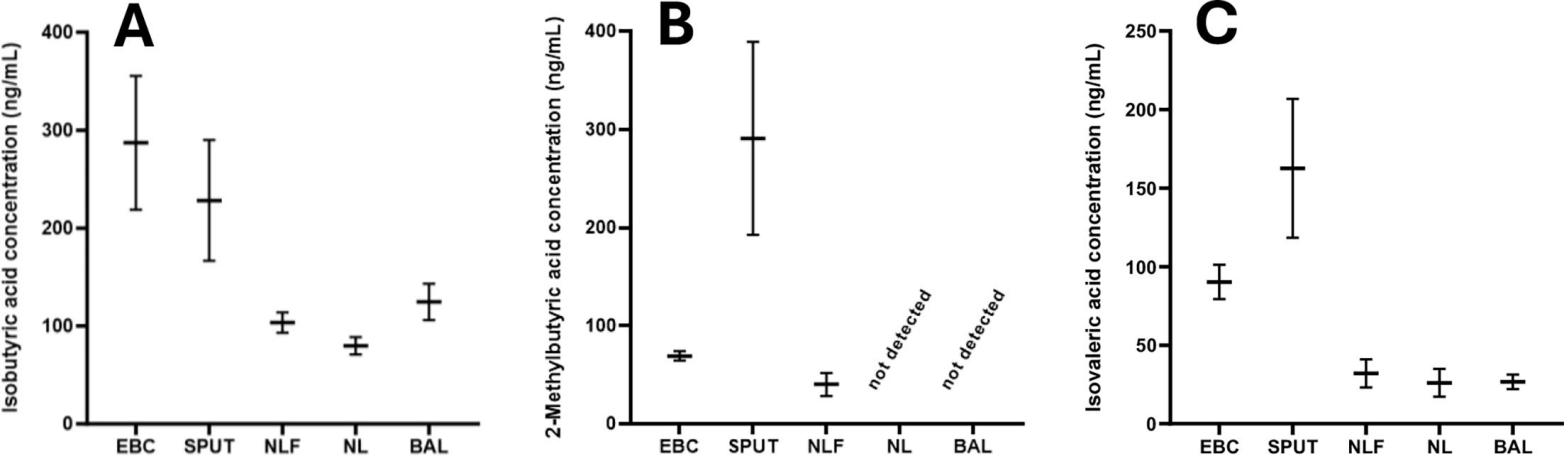
Concentrations of the branched-chain, short-chain fatty acids isobutyric acid (**A**), 2-methylbutyric acid (**B**) and isovaleric acid (**C**) within clinical respiratory samples. Horizontal bars represent the mean concentration with error bars visualising the standard error of the mean. BAL, bronchoalveolar lavage (*n*=42, 0 and 8); EBC, exhaled breath condensate (n of samples detected in isobutyric, 2-methylbutyric, isovaleric; 34, 19 and 32); SPUT, sputum (*n*=31, 17 and 23); NL, nasal lavage fluid (*n*=47, 0 and 6); NLF, nasal lining fluid (*n*=81, 4 and 14).

Additional patient respiratory samples were obtained from previous research investigations. These included EBC, sputum, nasal lavage fluid, BAL and nasal lining fluid from patients diagnosed clinically with respiratory conditions, smoking controls and healthy non-smoking controls. SCFAs were present at detectable levels in all sample types except for 2-methylbutyric acid, which was not detected for any patients in nasal lavage fluid or BAL samples. Overall, mean SCFA concentrations were elevated in EBC and sputum samples when compared with the other respiratory biosample types, with considerably higher concentrations reported in EBC/sputum for acetic acid (mean values ~2–9× higher), propionic acid (~11–30× higher), and butyric acid (~5–25× higher). All other acids showed moderately higher values (~1.2–6.2×) in EBC and sputum when compared with the other respiratory biosample types. The mean concentrations of the straight-chain fatty acids across the different biosample types are shown in Figure 4. Interestingly, when considering branched-chain fatty acids, which are known to be produced via protein fermentation, the concentrations were the highest in sputum for isovaleric acid and 2-methylbutyric acid and elevated but similar to EBC for isobutyric acid (Figure 5). These observations of the presence of branched-chain fatty acids in sputum require further investigations to better understand the origin(s) of acid production.

## Discussion

This work sought to assess the capability to measure SCFAs in EBC with a dynamic test to understand the relationship between circulating and exhaled SCFA content. In addition, it aimed to evaluate the potential to measure SCFAs in clinical respiratory biosamples, including EBC and a series of other relevant sample mediums. Acetic acid and propionic acid were observed to be at higher concentrations in EBC when compared with serum in baseline (i.e. pre-interventional) samples collected from healthy male participants. Contrastingly, butyric acid concentrations were shown to be comparable between EBC and serum. When SCFA-containing capsules were ingested by the participants intended to increase acute peripheral bioavailability, serum acetic and butyric acid concentrations were elevated within 60 min and returned to baseline values by 120 min in a manner that has been reported previously [34]. Propionic acid levels were not observed to increase post-supplementation as seen in previous work [34]. This observation is due to a lack of statistical power, as the 60 min timepoint for propionate had a raw *P* value of 0.026, which did not meet the critical value threshold for the Benjamini–Hochberg FDR (V_CRIT_=0.017). Interestingly, no changes in SCFA concentrations were observed in EBC samples for the ingested acids at any point across the 2 h observational period. Whilst this outcome may have been hindered by experimental power, high variation of EBC SCFA levels across participants meant that no effects were identified from the linear mixed models, a finding that contrasted all acids observed in serum. This suggested that SCFAs are not present in the lungs at levels that are proportional to serum concentrations and, therefore, EBC is not suitable to use as a surrogate sample type to inform on circulating SCFA content. Moreover, no correlative relationships for EBC and serum concentrations were reported. It would be expected that if SCFAs passively cross the blood–alveolar barrier, a degree of association between the two biofluid mediums would be seen. However, in order to fully understand the extent to which systemically available SCFAs enter the lung microenvironment, alongside potential inter-individual variation, the ingestion and measurement of isotopically labelled SCFA salts should be performed to provide specificity on the origin of the analysed acids. Furthermore, whilst we would not expect behaviours in SCFA ingestion and potential translocation into the lungs to differ between sexes, the male-only sample set investigation in this work restricts generalisability of the outcomes. In addition to the assessment of acute dynamics of SCFAs in serum and EBC, additional analyses were performed on a series of respiratory biosamples collected from patients diagnosed with respiratory conditions and healthy, non-smoking controls. This investigation identified a range of SCFAs (C2–C5 in straight and branched structures) that were detectable in EBC, sputum and nasal fluid, with 2-methylbutyric acid not detectable in nasal lavage fluid nor BAL. In general, elevated concentrations of SCFAs were reported in EBC and sputum, with a particularly interesting finding demonstrating that sputum is a rich sample medium for the detection of branched-chain SCFAs.

The presence of SCFAs within respiratory biofluids (i.e. EBC) has been attributed to be the result of transfer from the systemic circulation (originating in the gut) into the lungs, as well as via the production of SCFAs by the lung microbiota [30,40]. However, the relative contribution from circulation and lung microbial fermentation is not currently known. SCFAs are lipid-soluble molecules when protonated and, therefore, in this arrangement can passively transfer across cell membranes. However, when present in the circulating blood, >99% of SCFA molecules are present in their ionic form due to the pH of the environment (pH 7.35–7.45). This means that active transportation across cell membranes would be the predominant method for tissue uptake, reducing the likelihood of passive uptake at the blood–alveolar barrier [41]. Monocarboxylate transporters (MCTs) are a family of cell surface transporters which can facilitate the active uptake of SCFAs into tissues. MCTs are present on most cells that require substrate transport [41], with isoforms MCT1, MCT2 and MCT4 expressed on lung cell membranes and thus capable of transporting SCFAs into the lungs [42]. However, as MCTs are present across all major tissue types, the uptake of SCFAs is not exclusive to any individual system and, therefore, there may not be uniform uptake with respect to changes in circulating levels [42]. This active uptake by other tissues is a likely explanation that a relationship between EBC and serum SCFA levels was not observed when a dynamic test to change circulating levels was achieved through SCFA ingestion. Moreover, SCFAs are ligands for G-coupled protein receptors (GPR41, GPR43 and GPR109a), which are also present on cells around the body, such as hepatocytes, skeletal myocytes and immune cells, leading to further utilisation of SCFAs away from the respiratory system [7,43]. Indeed, previous work has shown that acute elevations in systemic SCFA levels have multiple physiologic and metabolic functions, including altered substrate oxidation, increased appetite hormone secretion and integration into biologically relevant substrates [11–13,44].

The identification that respiratory SCFA concentrations do not appear to be dynamically influenced by circulating SCFA content suggests that the abundance of SCFAs in EBC is predominantly influenced by lung microbial activity, albeit probable that systemic bioavailability provides some degree of contribution to lung SCFA content. The production of SCFAs as by-products from lung microbial metabolism may further explain the presence of increased concentrations of acetic acid and propionic acid in EBC when compared with serum, albeit that an accumulation of SCFAs in the lung over time is an additional and potential explanation. Recent advances in the understanding of the lung microbiome have identified that healthy gut and lung environments share similar core microbial properties. For example, the most abundant phyla within the lungs relate to those present in the gut, including *Bacteroidetes, Firmicutes* and *Proteobacteria*. The lung microbial profile is also influenced by the upper oral cavity, with similarities found between oral and nasal microbial communities [45]. These bacteria are substantial SCFA producers within the gastrointestinal environment [7] and, therefore, their presence within the respiratory system and upper oral cavity supports the notion that SCFA concentrations in respiratory biofluids are predominantly reflective of local production. However, the source for precursor molecules to ferment to SCFAs by the lung microbiome is not well established and, therefore, may not reflect the same characteristics seen within the large intestine, where dietary fibre is the main source for SCFA production. Interestingly, in this work, it was observed that sputum was a rich sample medium for the detection of branched-chain SCFAs. It is known that SCFAs can be produced via the breakdown of the mucus layer covering the large intestine [46], suggesting that the mucus layer within the lungs may provide a source of proteins for bacterial SCFA production. This concept is supported by the identification that branched-chain SCFAs were abundantly present in sputum samples. Sputum is a thick mucus produced in the lungs and airways which contains globular proteins [47]. The production of sputum can be increased in airway disease, with previous data identifying an increase in MUC5AC, a major airway gel-forming mucin, to be present in patients with COPD [48]. It has been previously shown that increases in the delivery of protein to the gut result in isovaleric acid and isobutyric acid production [10], indicating the likelihood that local fermentation of mucus proteins is leading to the elevated levels of branched-chain SCFAs observed in this work. Future work to understand if specific microbial taxa are present in the lungs which are known to be branched-chain SCFA producers would be of particular interest, alongside the amino acid profiling of the mucus to assess the likelihood of branched-chain SCFA production due to increased local availability of valine, leucine and/or isoleucine; unfortunately, these data were not available in the current clinical dataset.

One unexpected observation from this work included the large discrepancy in concentrations of propionic acid and butyric acid in EBC of the clinical samples when compared with the healthy participants recruited as part of the SCFA ingestion experiment (mean concentration in healthy vs clinical samples; propionic acid 1012 vs 5731 ng/ml and butyric acid 36 vs 984 ng/ml). It is not known the reason for these discrepancies, but the elevated levels seen in the clinical samples may be reflective of altered SCFA production and/or systemic transfer as a result of lung infection and/or injury. Indeed, *in vitro* and *in vivo* data from animal studies have demonstrated that increased production or delivery of propionate and butyrate, specifically, influenced the outcome to lung injury and infection via immune cell effects (e.g. neutrophil infiltration) [21,22,49]. Whilst this observation is of interest and warrants future investigation, the current data cannot reliably inform on these differences due to comparisons of samples collected via diverse methods, at different periods of time, and from a range of individuals.

In conclusion, concentrations of SCFAs in EBC are not related to those measured in the serum. The ingestion of SCFA salts to acutely elevate systemic concentrations was not reflected by increases in SCFA concentrations in EBC. These data suggest that SCFAs should not be used as a surrogate measure to inform on circulating levels using EBC as a minimally invasive procedure. In addition, as the relationships of dynamic changes were not comparable between serum and EBC, this suggests that exhaled SCFA content is reflective of local production via metabolism by the lung microbiota. This was further supported through the interesting observation that branched-chain SCFAs were abundant in sputum samples, potentially due to the fermentation of mucus proteins. Moreover, the concentrations of acetic acid and propionic acid were greater in EBC than in serum, which warrants further investigation to understand the sources of SCFAs and their biological relevance within the respiratory system. In addition, the elevated mean concentrations of propionic acid and butyric acid observed in clinical EBC samples when compared with healthy participants may signify altered SCFA production and/or delivery either due to or in response to respiratory infection and lung injury, although further studies are required to appropriately compare healthy vs respiratory disease patient samples. Overall, it was demonstrated that SCFAs can be detected across multiple clinical respiratory samples, including EBC, sputum, nasal lavage fluid, BAL and nasal lining fluid, providing multiple options for clinicians and researchers to investigate the influence of SCFAs in respiratory conditions.

**Clinical perspectives**

- Short-chain fatty acids (SCFAs) produced via microbial fermentation in the gut, and potentially within the lungs, have been shown to demonstrate protective effects in the lung microenvironment. Their presence in exhaled breath has resulted in further interest as to whether SCFAs in the lung originate from systemic circulation (i.e. produced in the gut and cross the blood–alveolar barrier) or are produced locally within the respiratory system.
- Oral SCFA supplementation increased serum SCFA levels in healthy participants, but a lack of corresponding rise or correlation with serum levels was observed in exhaled breath condensate. SCFAs were further detectable in various respiratory sample matrices, with particularly high levels, including branched-chain SCFAs, noted in sputum, suggesting local microbial fermentation within the lungs.
- The data suggest that exhaled SCFAs likely originate from lung microbiota rather than the gut, highlighting the potential for SCFAs as biomarkers of lung microbial activity. This could inform new diagnostic approaches and/or therapeutic strategies for lung microbiome monitoring/manipulation in respiratory diseases.

## Data Availability

Raw data values for measured SCFAs are available online at https://doi.org/10.17028/rd.lboro.29605925.

## Open Access

This article has been published open access under our Subscribe to Open programme, made possible through the support of our subscribing institutions, learn more here: https://portlandpress.com/pages/open_access_options_and_prices#conditional

## Abbreviations

BAL: bronchoalveolar lavage fluid
COPD: chronic obstructive pulmonary disease
CaPr: calcium propionate
EBC: exhaled breath condensate
FDR: false discovery rate
GC-MS: gas chromatography-mass spectrometry
GPR: G-coupled protein receptor
HPMC: hydroxypropyl methylcellulose
IS: internal standard
MCTs: monocarboxylate transporters
MTBE: methyl-tert-butyl ether
NaAc: anhydrous sodium acetate
NaBu: sodium butyrate
QC: quality control
SCFAs: short-chain fatty acids
